# The number of CCR5 expressing CD4+ T lymphocytes is lower in HIV-infected long-term non-progressors with viral control compared to normal progressors: a cross-sectional study

**DOI:** 10.1186/s12879-014-0683-0

**Published:** 2014-12-13

**Authors:** Hinta Meijerink, Agnes R Indrati, Reinout van Crevel, Irma Joosten, Hans Koenen, Andre JAM van der Ven

**Affiliations:** Department of Internal Medicine, Radboud University Medical Center, Route 456, Nijmegen, 6500 HB The Netherlands; Health Research Unit, Faculty of Medicine, Universitas Padjadjaran/Hasan Sadikin Hospital, Bandung, Indonesia; Laboratory of Medical Immunology, Department of Laboratory Medicine, Radboud University Medical Center, Nijmegen, The Netherlands

**Keywords:** HIV, CCR5, CXCR4, Elite controllers, T-lymphocytes, Regulatory T-cells, Memory T cells, Naïve T cells, HIV co-receptors

## Abstract

**Background:**

The HIV co-receptors CXCR4 and CCR5 play an important role in HIV infection and replication. Therefore we hypothesize that long-term non-progressors (LTNP) with viral control have lower expression of CCR5 and CXCR4 on CD4^+^ cells, specifically on memory T-lymphocytes since they are the primary target cells of HIV.

**Methods:**

In this cross-sectional study, we included five HIV-infected LTNP with viral control (CD4 > 750 cell/μl & HIV < 50 copies for ≥2 years), thirteen HIV-infected and seven HIV-uninfected individuals at Radboud UMC Nijmegen, the Netherlands. We determined the CCR5 and CXCR4 expression among CD4^+^ and CD8^+^ lymphocyte subsets; memory (CD45RO^+^), naïve (CD45RA^+^) cells and regulatory T-cells (CD4^+^CD25^high^FoxP3^+^). In addition, CCR5∆32 polymorphism is related with disease progression and was therefore determined using polymerase chain reaction.

**Results:**

The percentage of CCR5-expressing CD4^+^ cells of LTNP was comparable with healthy controls; whereas HIV-infected individuals showed more CCR5-expressing cells. This was observed in memory and naïve CD4^+^ cells, but not in regulatory T-cells. The mean fluorescence intensity of CCR5-expressing CD4^+^ cells was similar in all groups. All groups had comparable percentages of CXCR4-expressing cells. The mean fluorescence intensity of CXCR4-expressing cells was significantly higher in HIV-infected normally progressors in both memory and naïve CD4^+^ cells, but not in CD8^+^ cells. The CCR5∆32 polymorphism was not related to group.

**Conclusions:**

We show that HIV affects -directly or indirectly- the expression of CCR5 in CD4^+^ T-lymphocytes; yet this effect is not seen in LTNP with viral control. Avoiding upregulation of CCR5 could be an important method via which LTNP counteracts the effects of HIV and suppresses viral replication. Exploring how LTNP suppress the upregulation of CCR5 could be an important step for discovering new therapeutics.

**Electronic supplementary material:**

The online version of this article (doi:10.1186/s12879-014-0683-0) contains supplementary material, which is available to authorized users.

## Background

Human immunodeficiency virus (HIV) infects and destroys CD4^+^ T-lymphocytes. Resting CD4^+^ T-lymphocytes are a reservoir of HIV infection; subsets of these lymphocytes are differentially affected by HIV and subdividing these cells in memory and naïve cells can provide better understanding of their individual roles [1],[2]. Memory T-lymphocytes are preferentially targeted by HIV, whereas increased numbers of regulatory T-cells are associated with disease progression [3]. The density of the CD4 surface marker is associated with both HIV RNA viral load and disease progression [4]. In addition to CD4 receptors HIV requires a co-receptor, either CCR5 or CXCR4, to invade cells. In general, HIV isolated from newly infected individuals uses CCR5 and these “R5 variants” are detectable over the entire course of the HIV infection [5]. The X4 variants, that utilize CXCR4, are mostly detectible at a late stage of the disease and in only up to 50% of all patients [6]. Both co-receptors are expressed on leukocytes, but to different extents on different T-cell subsets [7],[8]. CXCR4 is predominantly found on resting, naïve T-lymphocytes, whereas CCR5 is expressed on mostly memory T-lymphocytes. Therefore activated CD4^+^CCR5^+^ T-lymphocytes are the primary target and an optimal subset for virus replication [2].

The number of circulating CD4 cells accurately reflects the extent of immunodeficiency in HIV-infected patients. Most HIV-infected patients exhibit a gradual decline in CD4 cells throughout the course of their infection; the rate of disease progression from asymptomatic HIV infection to AIDS varies between patients. Some HIV patients are able to maintain stable CD4 cell counts for an extended time and remain asymptomatic without ART for years after infection. These patients have been referred to as long-term non-progressors (LTNP). Unfortunately, the underlying mechanisms for the interindividual variability and slow progression of HIV are poorly understood. Understanding mechanisms that are associated with slow progression will help identify new targets for treatment and even prevention of HIV infection.

The HIV co-receptors CCR5 and CXCR4 could play a crucial role in the progression of HIV and non-progression among LTNP. A well-known polymorphism in the CCR5 gene, namely CCR5∆32 (a 32 base pair deletion in the CCR5 gene) interrupts the entrance of HIV into cells and individuals homozygous for this deletion are almost completely resistant to HIV infection [9]-[12]. And individuals who are heterozygous for the CCR5∆32 have shown slower progression of HIV [9],[13]-[15]. Also the expression of CCR5 and CXCR4 is, in addition to CD4, extremely important in the susceptibility of cells to HIV infection [16] and viral replication [17],[18]. Previous studies have shown that the expression of CCR5 increased with HIV disease progression, a phenomenon that is reversed by antiretroviral therapy (ART) [19]; indicating that CCR5 expression is important in disease progression. Not only the receptors, but also its ligands (MIP-1α/CCL3, MIP- 1β/CCL4 and RANTES/CCL5 for CCR5 and SDF-1/CXCL12 for CXCR4) have the ability to block HIV activity [12]. Unfortunately, only little information is available on the expression of CXCR4 and CCR5 on different subsets of T-lymphocytes, especially in relation to LTNP. Therefore, we have explored differences in the expression of CCR5 and CXCR4 in different subsets of T-lymphocytes in LTNP with viral control.

## Methods

### Study population

HIV seropositive, ART-naïve subjects above 18 years of age were included at the HIV-clinic of the Radboud University Nijmegen Medical Centre, the Netherlands. LTNP with viral control were defined as those with a CD4 cell count above 750 cells/μl and a HIV viral load below 50 copies/ml for at least 2 years [10],[20]. HIV-infected subjects that did not fit these criteria were classified as HIV-infected normal progressors, and HIV seronegative healthy individuals were used as controls. Blood samples of LTNP were always examined in parallel with samples from a healthy control donor and multiple HIV-infected normal progressors. Blood samples were used for standard hematologic blood parameters and flow cytometry.

### Ethics

This study was presented to the institutional review board of Radboud UMC in Nijmegen, the Netherlands. We received a waiver for blood collection of HIV infected individuals and approval for blood collection of healthy controls from the Medical Ethical Committee Nijmegen in the Netherlands. All participants, including healthy controls, were informed about giving blood samples for this specific study and provided written consent. During a routine appointment, nurses in the clinic collected the blood samples and provided these to us anonymously. None of the researchers had access to participants personal identifying data and all data was analysed anonymously.

### Flow cytometry

Cells were phenotypically analysed by five-colour flow cytometry (Coulter Cytomics FC 500, Beckman Coulter, Fullerton, CA) using Coulter Epics Expo 32 software. Both peripheral blood mononuclear cells (PBMC) and whole blood (after red cell lysis) were used for flow cytometric analysis. PBMC were isolated by density centrifugation on Ficoll-Hypaque (Pharmacia Biotech, Uppsala, Sweden). Red cell lysis of whole blood was performed according to the manufacturer’s instruction (BD PharmLyse, Becton Dickinson Biosciences, San Jose, USA). Cells were washed with phosphate-buffered saline (PBS) with 1% bovine serum albumin (BSA) before being incubated with fluorochrome- conjugated mAbs. After incubation for 20 min at room temperature, cells were washed twice to remove unbound antibodies and analysed. For cell surface staining, the following mAbs were used: CD25-PECy7 (BC96), CD127 PECy5 and PECy7 labelled (RDR5) from eBioscience (San Diego, USA); CD3-ECD (UCHT1) and CD4-ECD (SFCI12T4D11) from Beckman Coulter (San Diego, USA); CD4-PECy7 (RPA-T4), CD8-PECy7 (HIT8a), CD45-APC (HI30), CD14-APC (M5E2), CD19-PECy7 (HIB19), CD45RO-APC (UCHL1), CD45RA-APC (HI100), CXCR4-PE (12G5), CCR5-FITC (HEK/1/85a) from Biolegend (San Diego, USA). Appropriate isotype control mAbs were used for gate settings. Due to limited amount of filters per sample memory and naïve lymphocytes were identified using CD3 and CD4 in combination with CD45RO or CD45RA respectively. The live gate was set based on the forward angle light scatter (FSCs) and the side angle light scatter (SSCs). For intracellular staining for FoxP3 we used FoxP3-APC antibodies (PCP101; eBioscience). Before intracellular staining, cells were fixed and permeabilised using Fix and Perm reagent according to the manufacturer’s recommendations (eBioscience).

### DNA isolation and polymerase chain reaction

DNA was isolated from PBMCs by Puregene DNA isolation kits (Gentra Puregene blood kit, Qiagen, Hilden, Germany) according to manufacturer’s instruction. Briefly, cells were lysed overnight at 37°C before adding protein precipitation solution. DNA was purified using isopropanol, washed with 70% ethanol, and then diluted in 10 μl of DNA hydrolysation solution and incubated overnight at 37°C. Hydrolysation solution without DNA was used as a negative control. DNA concentrations were measured using Nanodrop and samples were stored at 4°C until analysis. To determine the 32 bp deletion in the CCR5 gene (CCR5∆32) we used polymerase chain reaction (PCR) with the following primers: forward primer 5′-ATCACTTGGGTGGTGGCTGTGTTTGCGTCTC-3′ and reverse primer 3′-GACGGCGACGAACAGTACCAGTAGACGATGA-5′, corresponding to bases 505–535 and 667–697 [21]. Genomic DNA from each individual was amplified in a total volume of 25 uL in a buffer containing 10 μM of each primer. The cycling conditions were: denaturation at 94°C for 5 min (1 cycle), followed 94°C for 60 sec and 70°C for 30 sec for 35 cycles. The CCR5∆32 polymorphism was detected with electrophoresis with 4% agarose gel stained with ethidium bromide. The normal allele size is 193 bp and the allele size for the deletion is 161 bp.

### Statistical analyses

**Figure 1:**
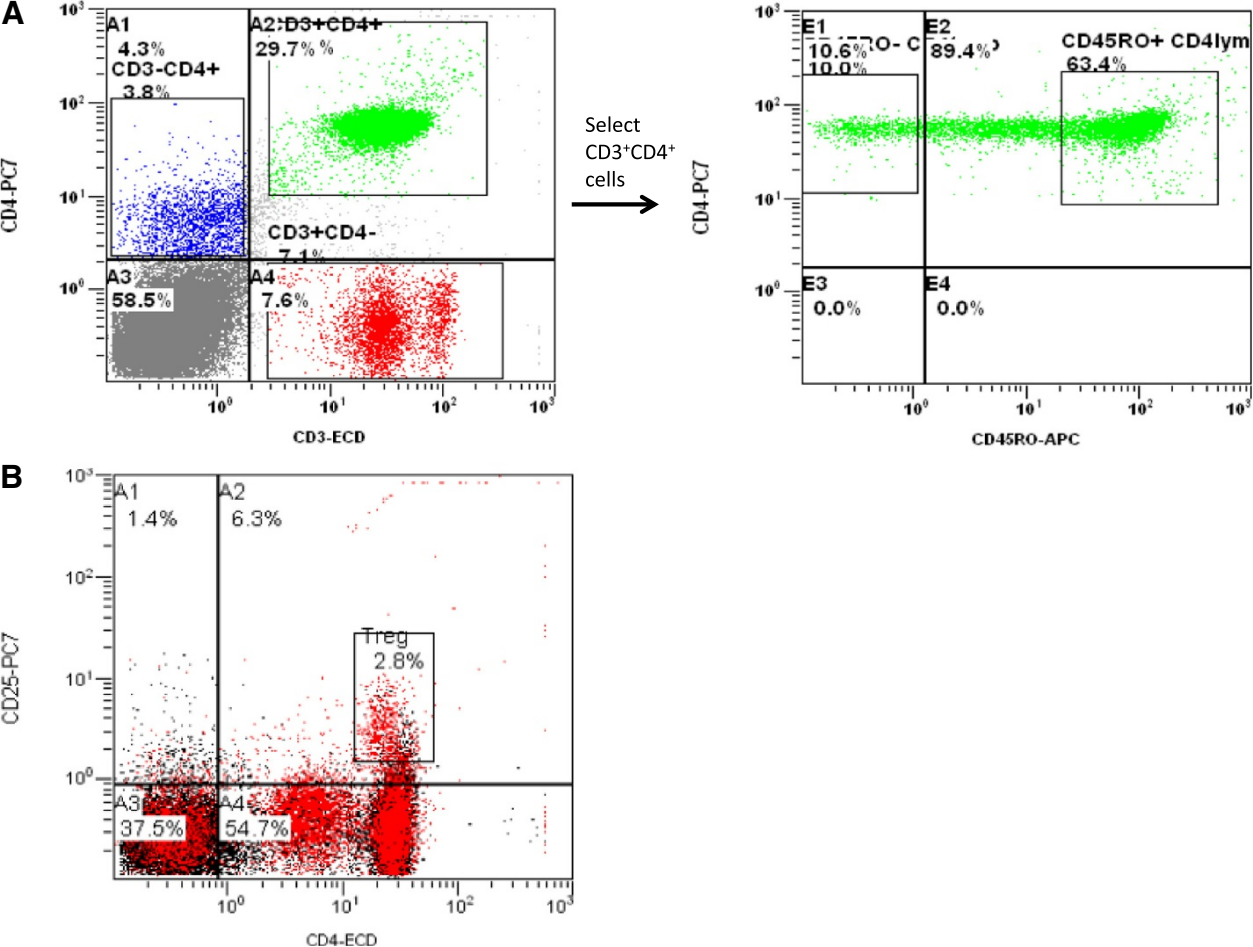
**Examples of how CD3** ^**+**^ **CD4** ^**+**^ **CD45RO** ^**+**^ **(A) and CD4** ^**+**^ **CD25** ^**high**^ **FoxP3** ^**+**^ **(B) cells were selected. A)** Cells were first gated on CD3^+^ CD4^+^ (green), selected cells were then split in CD45RO^+^ and CD45RO^-^ cells. Within CD45RO^+^ cells the expression of CCR5 and CXCR4 was determined. **B)** Regulatory T-cells were selected by gating on CD4^+^ and CD25^high^ cells, and only FOXP3^+^ cells were used for analyses.

We selected subsets of cells by first gating on live cells and subsequently on CD45+ cells. Within these gated cells we defined the subsets as follows: monocytes: CD14^+^; B-cells: CD19^+^; T-lymphocytes: CD3^+^. In the CD3^+^ cells we gated on either on CD4^+^ or CD8^+^ cells. Further, within these CD4^+^ or CD8^+^ cells we identified CD45RA^+^ (naïve), CD45RO^+^ (memory) and CD25^high^FoxP3^+^ (regulatory). In Figure 1A we give an example of how memory cells were selected. First we selected all CD45^+^ cells and plotted CD3 and CD4 markers in a scatterplot (left), then we selected all CD4^+^ CD3^+^ cells and plotted CD4 and CD45RO markers in a second scatterplot (right). In this plot we identified memory cells as CD45RO^+^ CD4^+^ cells. In Figure 1B we plotted CD25 and CD4 markers and identified regulatory T cells as being expressing CD25 high, CD4 and FoxP3. For all cell subtypes, expression of HIV co-receptors CCR5 and CXCR4 was measured as proportion (%) of positive cells, as well as mean fluorescence intensity (MFI) of positive cells. The Kruskal Wallis and Mann Whitney analyses were used to compare differences between groups. The level of significance was set at 10%. All statistical analysis was performed using the Statistical Product and Services Solutions package version 18.0 and GraphPad Prism version 5.0.

## Results

We included five HIV-infected LTNP with viral control, thirteen HIV-infected normal progressors and seven healthy controls. None of the HIV-infected subjects took ART at time of blood collection or previously. The characteristics of the subjects are shown in Table 1; as expected the median CD4 cells were higher and the viral load was lower in HIV-infected LTNP than in normal progressors. HIV was diagnosed a median of eight years (range: 3–14 years) for LTNP and a median of three and a half years (range: 1–7 years) for other HIV infected individuals.

**Table 1:** **Characteristics of ART-naïve HIV-infected individuals and controls devided per group (n = 25)**

	HIV-infected		Healthy controls
	*LTNP*	*Normal progressors*	
Number	5	13	7
Median age, years	49	39	41
Median CD4 cells, cells/μl	1240	565	n.a.
Median Log (viral load)	1.7	4.3	n.a.

ART: antiretroviral therapy; HIV: human immunodeficiency virus; LTNP: long-term non-progressors; IQR: interquartile range.

Differences in cell type distribution between groups were most pronounced for CD4^+^ subtypes (Table 2). As expected, HIV-infected normal progressors had significantly lower CD4^+^ T-cell counts compared to LTNP and healthy controls (Table 2). This difference was found for all CD4^+^ subsets: memory (CD45RO^+^), naïve (CD45RA^+^) and regulatory (CD25^high^FoxP3^+^ cells) T-lymphocytes.

**Table 2:** **Cell types and expression of CCR5 and CXCR4 for HIV-infected and uninfected individuals**

Cell type	Control	LTNP	HIV	p-value
Total white blood cells, n (*10 ^**6**^ **/L)**	6043	7060	5846	0.214
CD14+ of CD45+ cells	9.0	8.2	10.1	0.186
CD3+ of CD45+ cells	24.7	29.9	30.7	0.188
CD8 + CD3+ of CD45 + cells	7.2	11.5	19.1	0.003
*CCR5+*	50.6	43.1	57.8	0.161
CXCR4+	92.2	97.4	95.1	0.590
CD4 + CD3+ of CD45 + cells	14.94	16.62	8.99	0.018
*CCR5+*	22.5	16.9	30.0	0.014
CXCR4+	70.2	83.7	71.9	0.185
CD45RO+ of CD3 + CD4+ cells	49.6	45.6	52.6	0.360
*CCR5+*	40.1	30.5	44.4	0.065
CXCR4+	45.8	70.6	57.7	0.191
CD45RA+ of CD3 + CD4+ cells	45.4	52.6	44.5	0.396
*CCR5+*	6.4	8.5	18.6	0.018
CXCR4+	91.6	94.6	90.3	0.595
CD25^high^FoxP3+ of CD3 + CD4 + cells	6.5	4.8	5.4	0.091
*CCR5+*	49.1	41.9	49.4	0.790
CXCR4+	97.9	98.2	95.6	0.712

Data are given as percentage unless stated otherwise.

Groups were compared with Kruskal-Wallis analyses.

HIV: human immunodeficiency virus; LTNP: long-term non-progressors; Control: HIV uninfected healthy controls.

**Figure 2:**
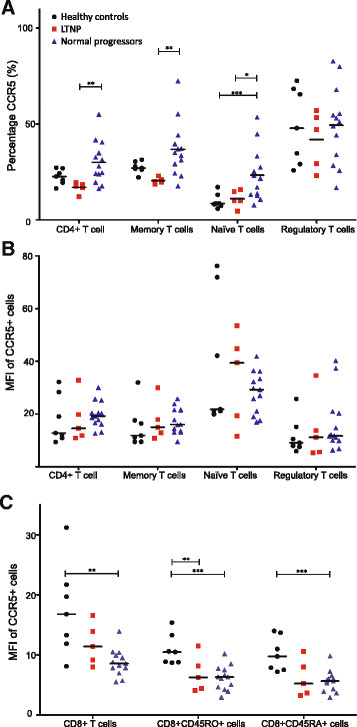
**The expression of CCR5 on various subsets of T-lymphocytes.** The percentage **(A)** and mean fluorescence intensity (MFI) **(B)** of CCR5 on several subsets of CD4^+^ T-lymphocytes and the MFI of CCR5 **(C)** on CD8^+^ T-lymphocytes for healthy controls, long-term non-progressors (LTNP) and HIV-infected normal progressors. Subsets of are defines as: memory T-cells: CD3^+^CD4^+^CD45RO^+^ cells; naive T-cells: CD3^+^CD4^+^CD45RA^+^ cells; regulatory T-cells: CD4^+^CD25^high^FoxP3^+^ cells. P-values: * < 0.100; ** <0.050; *** <0.001.

Expression of the HIV co-receptor CCR5 on CD4^+^ T-cells was similar in LTNP and healthy controls; both groups showed lower CCR5 expression compared to HIV-infected normal progressors (Figure 2A). When CD4-cell subsets were analysed, this difference was observed in memory and naïve T-lymphocytes (p-values: 0.007 and 0.046 respectively), but not in regulatory T-cells (p = 0.443) (Figure 2A). CD8^+^ T-lymphocytes did not show a difference in CCR5 expression.

The MFI of CCR5-expressing cells in CD4^+^ T-lymphocytes was slightly higher in LTNP and HIV-infected individuals (Figure 2B; not significant). In all CD8^+^ T-lymphocyte subsets the MFI of CCR5-expressing cells was lower in HIV-infected normal progressors compared to healthy controls (Figure 2C).

**Figure 3:**
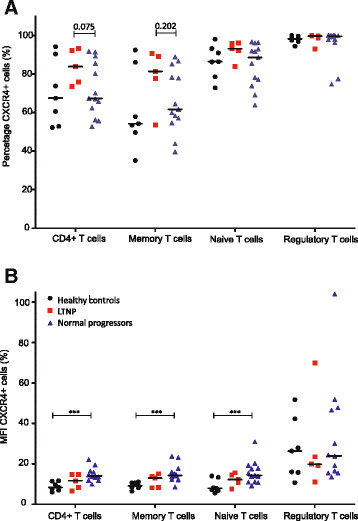
**The expression of CXCR4 on various subsets of T-lymphocytes.** The percentage **(A)** and mean fluorescence intensity **(B)** of CXCR4 on several subsets of CD4^+^ T-lymphocytes for healthy controls, long-term non-progressors (LTNP) and HIV-infected normal progressors. Subsets of are defines as: memory T-cells: CD3^+^ CD4^+^CD45RO^+^ cells; naïve T-cells: CD3^+^ CD4^+^ CD45RA^+^ cells; regulatory T-cells: CD4^+^ CD25^high^FoxP3^+^ cells. P-values: * < 0.100; ** <0.050; *** <0.001.

**Figure 4:**
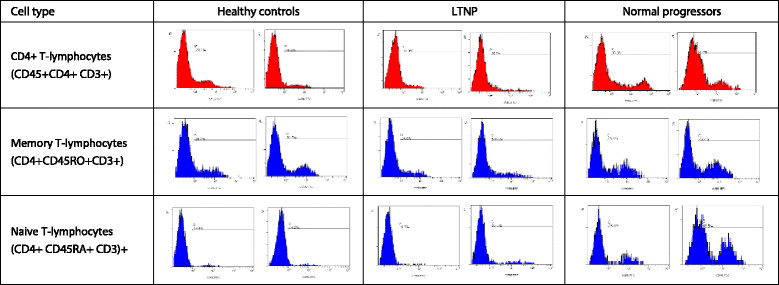
**Representative plots of the expression of CCR5 on various cell types for different groups.** Per group, namely healthy controls, long-term non-progressors (LTNP) and HIV-infected normal progressors, we show histograms from two representative subjects for the expression of CCR5.

**Figure 5:**
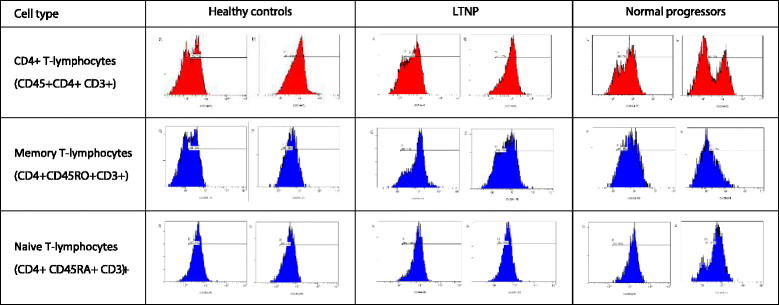
**Representative plots of the expression of CXCR4 on various cell types for different groups.** Per group, namely healthy controls, long-term non-progressors (LTNP) and HIV-infected normal progressors, we show histograms from two representative subjects for the expression of CXCR4.

In LTNP, the proportion of CD4^+^ T-cells that expressed CXCR4 was similar (Figure 3A). However, the MFI of CXCR4-expressing cells was significantly higher in HIV-infected normal progressors than healthy controls (Figure 3B). In all subsets of CD8^+^ T-lymphocytes the MFI of CXCR4-expressing cells was comparable between all groups. The differences between groups can be observed by two representative plots for all cell types for CCR5 (Figure 4) and CXCR4 (Figure 5).

The CCR5∆32 polymorphism in the CCR5 receptor may influence the expression of CCR5 [17]. Therefore we examined whether this polymorphism is more prevalent among the LTNP in this study. In total three people were heterozygote for the CCR5∆32 polymorphism (12%), one of which was LTNP (1/5) and two were HIV-infected normal progressors (2/12). No statistical difference was found between the prevalence of CCR5∆32 between LTNP and HIV-infected normal progressors (p = 0.813). In addition, the CCR5∆32 polymorphism was not (significantly) associated with the percentage nor the MFI of CCR5-expressing and CXCR4-expressing cells.

## Discussion

In this study we show that HIV infection is -directly or indirectly- associated with the expression of CCR5, but not CXCR4, in specific CD4^+^ T-lymphocyte subsets. This effect is most notable in HIV-infected normal progressors, but barely observed in LTNP with viral control.

CCR5 and CXCR4 are crucial for HIV invasion and they are expressed on various cell types, including hematopoietic cells and several cells in the central nervous system [7],[12],[17],[22]-[27]. The expression of CCR5 can be affected by HIV and we show, in line with other studies, that HIV-infected individuals have an increased CCR5 expression on CD4^+^ T-lymphocytes [17],[28]-[31]. Longitudinal studies show that the percentages of CCR5-expressing CD4^+^ cells increases slightly over time [17], while HAART reduces the percentage of CCR5-expressing cells [28],[29],[31],[32]. This suggests, that indeed HIV increases the expression of CCR5 on CD4^+^ T-lymphocytes and that this effect is reversible. In contrast, we found no association between HIV and the surface density of CCR5, expressed by MFI. This also accords with earlier observations, which showed that surface density of CCR5 is stable over time [18],[33].

Recently, studies suggest that subtle genetic differences of HIV are associated with non-progression. HIV derived from long-term non-progressors has shown a reduced cell entry *in vitro* [1],[34]-[37]. Also genetic differences in the host may play a role; the CCR5∆32 polymorphism is associated with lower numbers of CCR5-expressing CD4^+^ T-cells [17] and slower progression of HIV [9],[13]-[15]. However, the CCR5∆32 polymorphism is unrelated to the elevated expression of CCR5 in HIV-infected individuals, both in our study and that of others [15],[17],[30]. Despite these contradictory results, meta-analyses of published cohorts associate the CCR5∆32 with lower viral loads, decreased risk of progression to AIDS and lower mortality rate [13],[38]. In addition slow progression could be associated with pathways including specific cellular proteins, such as restriction factors, APOBEC3, TRIM5, and Tetherin. These molecules act at several key steps of the HIV lifecycle and can avert viral infection or replication in a cell-specific way. As they control HIV infection, it is possible that genetic alterations or levels of expression are related to differences in HIV progression [39].

We demonstrate that in LTNP with viral control the expression of CCR5 is lower on both memory (CD45RO^+^) and naïve (CD45RA^+^) CD4+ T-lymphocytes, but not on regulatory T-cells (CD4^+^CD25^high^FoxP3^+^). Only few studies examined co-receptors expression among specific HIV-infected patients, such as elite controls and LTNP, in combination with specific cell subsets [24],[40]. Almeida *et al.* presents the CCR5 expression in dendritic cells of HIV-infected patients, including long term non-progressors. They found lower expression of CCR5 in all HIV-infected patients [40]. Other studies showed lower expression of CCR5 CD4^+^ T lymphocytes in HIV controllers [24],[33]. Since CCR5 expression regulates whether HIV can infect target cells [41] and CCR5 down-regulation may contribute to the low levels of infection in LTNP.

Several studies analysed the association between the expression of CCR5 and disease progression, such as time to AIDS, CD4 cell decline or serum viral load levels. In both humans and animals, higher amounts of circulating CD4^+^CCR5^+^ T-lymphocytes are associated with faster disease progression, higher viral loads and lower CD4 cell counts [17],[18],[30],[33],[42],[43]. Our findings further support an association between CCR5 expression and HIV progression. In addition, Yang *et al.* show that in mangabeys infected with SIV (simian immunodeficiency virus) the CCR5 expression is significantly lower on all subsets of cells in slow progressors compared to fast progressors; which supports our findings [43]. The downregulation of CCR5 in LTNP can be influenced by higher levels of chemokines, which activates receptor internalisation and thereby reduces CCR5 expression [12],[44]. Also the amount of regulatory T cells are associated with disease progression; in line with other studies the amount of regulatory T cells was higher in HIV normal progressors than in LRNP with viral control [13].

In our study, the amount of CXCR4-expressing cells was comparable between groups, but the MFI of CXCR4-expressing CD4^+^ T-lymphocytes was significantly increased in HIV-infected individuals. Literature on the expression of CXCR4 is inconclusive; some studies revealed no differences [29],[42], whereas others observed reduced CXCR4 expression on CD4^+^ cells [17],[28],[30],[31], CD8^+^ cells [28],[30],[31], CD14^+^ cells [30] and natural killer cells [32]. CXCR4 is of more importance during later stages of infection [42] and therefore CXCR4 is probably less important in LTNP. In addition, all HIV-infected subjects in our study were asymptomatic and therefore the expression of CXCR4 might be affected by HIV at a later stage. Our study shows that LTNP with viral control tend to have a slightly higher number of CXCR4 expressing cells, albeit not significant, and also previous studies showed that there is a correlation between disease stage and CXCR4 expression of CD4^+^ T-lymphocytes; with healthier patients expressing more CXCR4 [30].

## Conclusion

In conclusion, the expression of CCR5 on CD4^+^ T-lymphocytes could be important in LTNP to counteract the effects of HIV and suppress viral replication. However, since this is a cross-sectional study the decreased expression of CCR5 could also be a consequence of the suppressed viral load in LTNP. Exploring whether LTNP suppress the upregulation of CCR5 could be an important step for discovering new therapeutics. Currently, CCR5 specific blockers, such as Maraviroc, are already used effectively in HIV treatment regimes, showing the importance of CCR5 in HIV suppression.

## Authors’ original submitted files for images

Below are the links to the authors’ original submitted files for images. Authors’ original file for figure 1 Authors’ original file for figure 2 Authors’ original file for figure 3 Authors’ original file for figure 4 Authors’ original file for figure 5
