# Supplementary figures and images for: The number of CCR5 expressing CD4+ T lymphocytes is lower in HIV-infected long-term non-progressors with viral control compared to normal progressors: a cross-sectional study
Source: BMC Infect Dis. 2014 Dec 13;14:683. doi: 10.1186/s12879-014-0683-0 (PMC4271479; doi:10.1186/s12879-014-0683-0)

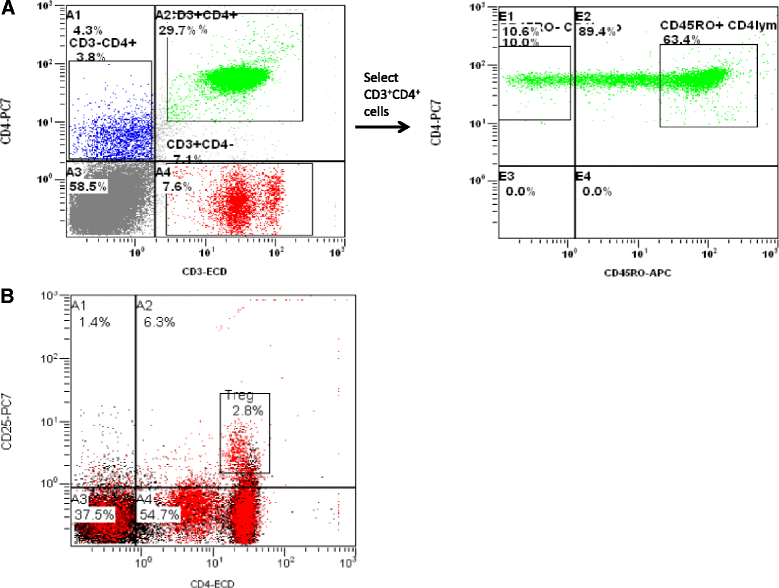

Supplement: Supplementary file 1 — Authors’ original file for figure 1 [file 12879_2014_683_MOESM1_ESM.gif]

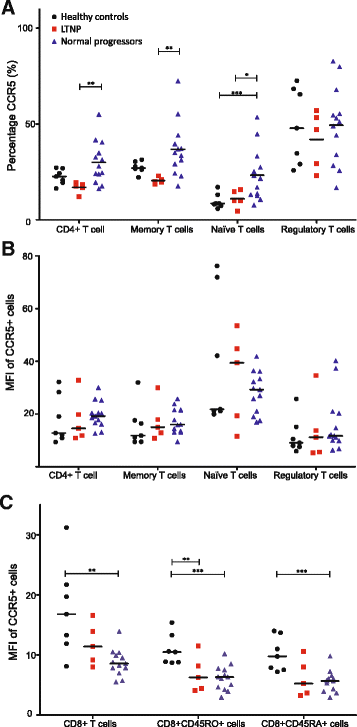

Supplement: Supplementary file 2 — Authors’ original file for figure 2 [file 12879_2014_683_MOESM2_ESM.gif]

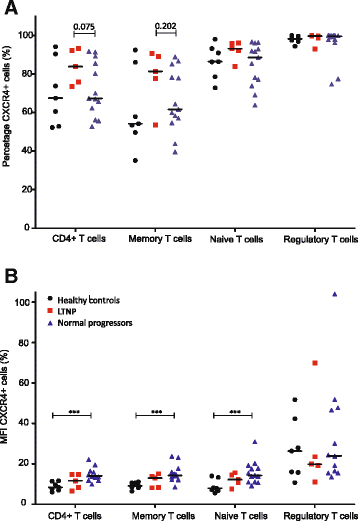

Supplement: Supplementary file 3 — Authors’ original file for figure 3 [file 12879_2014_683_MOESM3_ESM.gif]

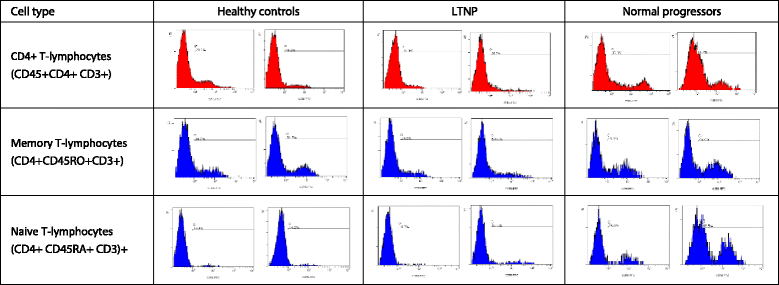

Supplement: Supplementary file 4 — Authors’ original file for figure 4 [file 12879_2014_683_MOESM4_ESM.gif]

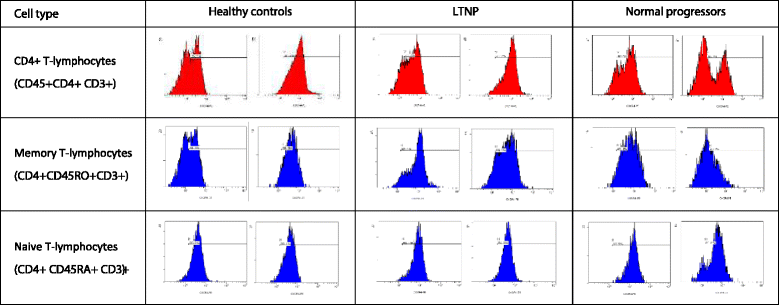

Supplement: Supplementary file 5 — Authors’ original file for figure 5 [file 12879_2014_683_MOESM5_ESM.gif]
